# Autophagy Dysregulation in Diabetic Kidney Disease: From Pathophysiology to Pharmacological Interventions

**DOI:** 10.3390/cells10092497

**Published:** 2021-09-21

**Authors:** Claudio D. Gonzalez, María Paula Carro Negueruela, Catalina Nicora Santamarina, Roxana Resnik, Maria I. Vaccaro

**Affiliations:** 1 Instituto de Bioquimica y Medicina Molecular Prof. Alberto Boveris (UBA-CONICET), Facultad de Farmacia y Bioquimica, Universidad de Buenos Aires, Buenos Aires C1113 AAD, Argentina; claudiodanielg@gmail.com (C.D.G.); rresnik@docente.ffyb.uba.ar (R.R.); 2 Instituto Universitario del Centro de Educacion Medica e Investigacion Clinica (IUC-CEMIC-CONICET), Facultad de Medicina, Instituto Universitario CEMIC, Buenos Aires C1430 EFA, Argentina; mpaulacarro@gmail.com (M.P.C.N.); nicoracatalina@gmail.com (C.N.S.)

**Keywords:** diabetic kidney disease, autophagy, pharmacological treatment, podocytes, proximal-tubular cells, metformin, SGLT2 inhibitors, GLP1 receptor agonists, renin-angiotensin-aldosterone system inhibitors (RAASi)

## Abstract

Diabetic kidney disease (DKD) is a frequent, potentially devastating complication of diabetes mellitus. Several factors are involved in its pathophysiology. At a cellular level, diabetic kidney disease is associated with many structural and functional alterations. Autophagy is a cellular mechanism that transports intracytoplasmic components to lysosomes to preserve cellular function and homeostasis. Autophagy integrity is essential for cell homeostasis, its alteration can drive to cell damage or death. Diabetic kidney disease is associated with profound autophagy dysregulation. Autophagy rate and flux alterations were described in several models of diabetic kidney disease. Some of them are closely linked with disease progression and severity. Some antidiabetic agents have shown significant effects on autophagy. A few of them have also demonstrated to modify disease progression and improved outcomes in affected patients. Other drugs also target autophagy and are being explored for clinical use in patients with diabetic kidney disease. The modulation of autophagy could be relevant for the pharmacological treatment and prevention of this disease in the future. Therefore, this is an evolving area that requires further experimental and clinical research. Here we discuss the relationship between autophagy and Diabetic kidney disease and the potential value of autophagy modulation as a target for pharmacological intervention.

## 1. Introduction

Diabetes mellitus (DM) is a metabolic disease characterized by chronic hyperglycemia [1]. There is a worldwide increase in the prevalence of this pathology, accounting for approximately 10% of the population over the age of 20 (close to 460 million people) in 2019. It is forecasted to affect 11% (700 million) by 2045 [2]. Chronic hyperglycemia may arise from a lack of insulin production (type 1 diabetes mellitus, T1DM) or to an exaggerated resistance to the cellular effects of insulin, accompanied by a decline in insulin production (type 2 diabetes mellitus, T2DM; the most frequent form). This condition is associated with macro-and microvascular complications. The kidney is the main target for microvascular damage in both T1DM and T2DM [3]. Near one out of two adults with T2DM and one out of three adults with T1DM will develop DKD.

Autophagy is an elemental cellular mechanism that transports intracytoplasmic components to lysosomes to preserve cellular function and homeostasis [4]. Considered a self-protection response to stress, it degrades and recycles endogenous materials to maintain energy levels [5]. Moreover, it operates as a quality assurance system through the elimination of injured or old cells in anticipation of further damage that contributes to human diseases [6]. For instance, in response to oxidative stress, autophagy increases to remove oxidatively damaged organelles, such as mitochondria [7]. However, dysregulated, or exaggerated autophagy may lead to autophagy impairment, which has been linked to a range of acute, chronic, age-related, or degenerative diseases [8]. 

There are three conventional types of autophagy: microautophagy, chaperone-mediated autophagy, and macroautophagy; however, the latter has a significant capacity to degrade entire organelles and large protein aggregates [9]. Macroautophagy is a multistep process whereby a double-membrane vesicle, called autophagosome, sequesters the cytoplasmic cargo. This eventually fuses with lysosomes to form autolysosomes, resulting in cargo degradation [10]. Additionally, microautophagy involves the intake of small macromolecules by invagination or protrusion of either the lysosome or the mature endosome [11]. Lastly, chaperone-mediated autophagy describes a more selective approach that targets specific proteins and transfers them to the lysosome for degradation [9]. 

The rate of autophagy in a determined tissue is biologically relevant. However, autophagosome density at any specific cell results from macroautophagy activation and intensity, as well as the rate of the terminal stage processes driving autolysosome formation. This ‘autophagic flux’ is of key importance when evaluating the contribution of macroautophagy to cell metabolism, homeostasis, and survival [12]. Autophagic flux is a measure of autophagic degradation activity [13]. 

The accumulation of damaged proteins and organelles due to hyperglycemia and other diabetes-related metabolic changes is highly associated with the development of diabetic nephropathy. Recent studies have suggested that both podocytes and proximal tubular cells exhibit altered autophagy activity under diabetic conditions. In addition, other non-conventional autophagic processes play a role in the pathophysiology of diabetic kidney disease (DKD).

A non-canonical type known as secretory autophagy (SA), which involves the release of proteins that lack a signal peptide, has been recently described [14]. Generally, cells secrete proteins to the extracellular compartment through exocytosis [15] following a specific pathway led by an N-terminal signal peptide. This permits the entry of proteins into the endoplasmic reticulum (ER), the Golgi apparatus, the secretory vesicles and finally, their release into the extracellular space by the fusion of membrane vesicle with the plasma membrane [16]. In the absence of a signal peptide, the non-conventional process of autophagy known as SA can cause the secretion of the protein [17,18,19]. Alterations in SA can accompany immunoinflammatory processes linked partly to renal kidney damage in diabetic nephropathy.

Therefore, diabetic nephropathy is associated with alterations in the different types of autophagic processes. Nevertheless, macroautophagy (hereafter referred to as autophagy) dysregulation is the best studied in the pathophysiology of DKD. These alterations may be clinically relevant in terms of disease prognosis and response to therapy.

## 2. Molecular Basis of Autophagy 

**Figure 1:**
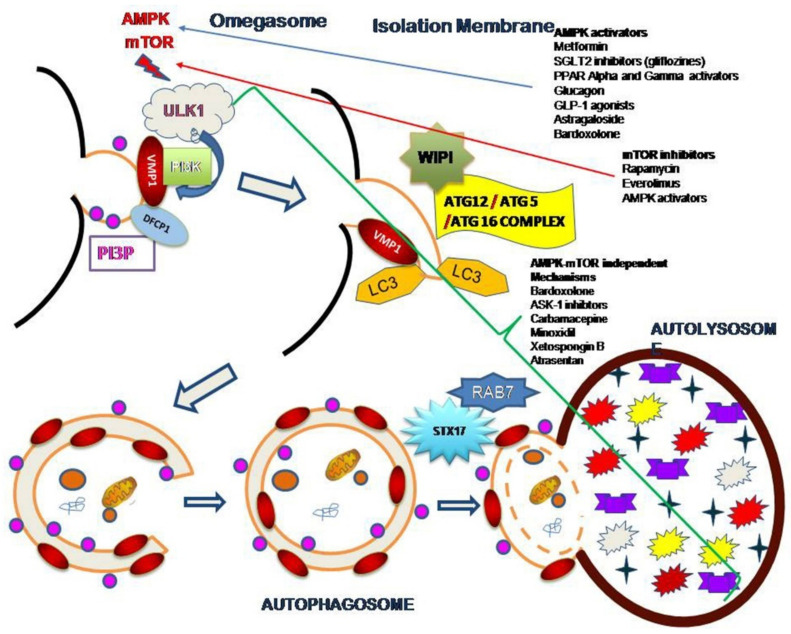
Autophagy activators in renal tissue. Putative AMPK-mTOR-dependent and -independent mechanisms are pointed on the schematic overview of the authophagic pathway. AMPK stimulators and mTOR inhibitors induce autophagosome biogenesis, while AMPK-mTOR-independent mechanisms can modulate the whole process along the autophagy flux. ULK1: unc-51-like kinase 1; VMP1: Vacuole membrane protein 1; PI3KC3 Phosphatidylinositol 3-kinase C3; FYVE. FYVE zinc finger domain; DFCP1, Double FYVE containing protein 1; PI3P Phosphatidylinositol 3-phosphate; GLP1, Glucagon like peptide 1; ATG, Autophagy-related gen or protein: ATG12-ATG5 complex; LC3, Microtubule-associated proteins 1A/1B light chain 3B (also known as MAP1LC3B); WIPI, WD repeat domain phosphoinositide-interacting protein; ATG12-ATG5-ATG16 complex; STX17 Sintaxin 17; RAB7 Ras-related protein 7; mTOR, mammalian Target of Rapamycin; SGLT2, Sodium Glucose Cotransporter 2; AMPK, AMP-activated Kinase.

The mammalian (or mechanistic) target of rapamycin (mTOR) and AMP kinase (AMPK) are two critical molecules associated with the core molecular mechanisms linked with autophagy regulation. The latter acts as an energy sensor. It regulates cell metabolism and energy homeostasis. The former integrates growth factors and the nutrient signals that regulate cell growth [20]. The biology of autophagy also implicates the autophagy related (ATG) genes and proteins. mTOR is usually responsible for the inhibition of autophagy, whereas AMPK acts as an upstream regulator of the process [21] (Figure 1).

The first step in the biogenesis of autophagosomes involves the phosphorylation of ULK1 that the activation of AMPK or the inhibition of mTOR signaling produces [22,23,24]. Once ULK1 becomes active, subsequent phosphorylation of the P13KC3 complex occurs. This complex forms under the action of BECN1, ATG14/15, and Vps34, which are crucial members of autophagosome formation [20].

The vacuole membrane protein 1 (VMP1) is a transmembrane protein whose expression is rapidly induced in the kidney under experimental hypoxia in vivo [25] and is implicated in the activation of autophagy [26]. Under certain biological conditions, after ULK1 activation, VMP1 interacts with the BH3 domain of BECN1 through its ATG domain, resulting in the recruitment of the P13KC3 complex to the autophagosome membrane. Thus, VMP1 plays an important role in the correct organization of ATG conjugation systems involved in the initial steps of autophagosome formation [20,27] (Figure 1). 

The phosphorylation of ATG12 and LC3 by the BECN1-PI3KC3 complex ensue from these events, resulting in the correct recognition of P13P [28]. The double FYVE domain-containing protein, DFCP1, then recognizes PI3P on the omegasome structure [29]. Hence, we consider DFCP1, a PI3P effector, as a marker of omegasome formation [30]. The resultant ATG12-ATG5 complex then recruits LC3 to the autophagosomal membrane. The ATG16L protein arbitrates this step, through its interaction with ATG5 to form the ATG12-ATG5-ATG16L complex [31]. The WIPI protein then recruits this complex to the isolation membrane [32].

The LC3 protein plays a major role in autophagy because of its involvement in elongation, maturation, and fusion of the autophagosome-lysosome [33,34]. The lipidated LC3 (LC3B) forms part of both sites of the autophagosome isolation membrane. It is considered a marker of autophagosomes, along with other ATG8 family proteins [35]. When the autophagosome presents to the lysosome, the hydrolases degrade the LC3 reservoir; the ATG4b cleaves the LC3 confined in the external membrane then recycles it [36]. Finally, the fusion between the autophagosome and the lysosome relies on the HOPS complex through STX17 and RAB7 [37,38]. Recent studies have implied the involvement of ATG14 in this process through its interaction with the SNARE protein STX17 [39] (Figure 1).

As mentioned before, the secretion related to autophagy may also play a role in the development, progression, and prognosis of DKD. Unconventional autophagy-mediated secretory pathways have shown biological relevance in recent years [40] due to their participation in the release of several aggregation-prone proteins [41]. Despite its lack of elucidation, the mechanism involving unconventional protein secretion relates to ATG-proteins [16], in that both canonical and non-canonical pathways may share their machinery [42]. Moreover, autophagy has shown association with exosome secretion [41]. Studies have proposed that the exosome secretion pathway begins with early endosomes that mature into multivesicular bodies (MBVs) inside the endosomal lumen. The latter may fuse with the plasma membrane to release its vesicles [43]. Furthermore, evidence suggests that ATG5, an essential protein for canonical autophagy, also participates in exosome production [44].

Autophagy also mediates secretion of the pro-inflammatory cytokine interleukin-1β (IL-1β). The LC3B-positive carriers sequester IL1β from the cytosol and fuse with the plasma membrane to release this cytokine through a secretory autophagy process [16]. The role of autophagy in pro-inflammatory mediator secretion is not restricted to inflammasome substrates. It extends to the secretion of other cytosolic inflammatory proteins lacking leader peptides that play a role in the progression of DKD. Insulin-degrading enzyme (IDE) is a zinc metalloprotease responsible for the cleavage and further inactivation of insulin [44,45], as well as other bioactive peptides such as glucagon, amylin, somatostatin, endorphins, and the beta-amyloid peptide (Ab). Highly expressed in renal tissues, IDE lacks any secretory signal sequence; therefore, it is not released through the classical exocytic pathway. For this reason, a non-conventional pathway secretes less than 10% of this protein, following a C-terminal Sly sequence that impedes its breakdown into lysosomes. The IDE protein acts as a modulator of inflammatory stress. Research has proposed that IDE can block NF-κB, a well-recognized transcription factor that regulates the genes responsible for several pro-inflammatory responses initiated at several stages of DKD progression [46]. Alterations in SA may affect IDE secretion and contribute to the upregulation of the pro-inflammatory environment that characterizes the evolution of DKD.

## 3. Autophagy and Kidneys

While autophagy appears to be expendable for kidney development, it appears to be essential for its integrity and proper functioning [47]. Reportedly, autophagy plays an important protective role in the kidneys by preventing the fibrosis and inflammation attributed to DKD [43]. Studies on rodents with streptozocin (STZ)-induced diabetes have shown that in diabetic nephropathy there is an early inhibition of autophagy in podocytes and proximal tubule epithelial cells (PTEC) [48].

**Figure 2:**
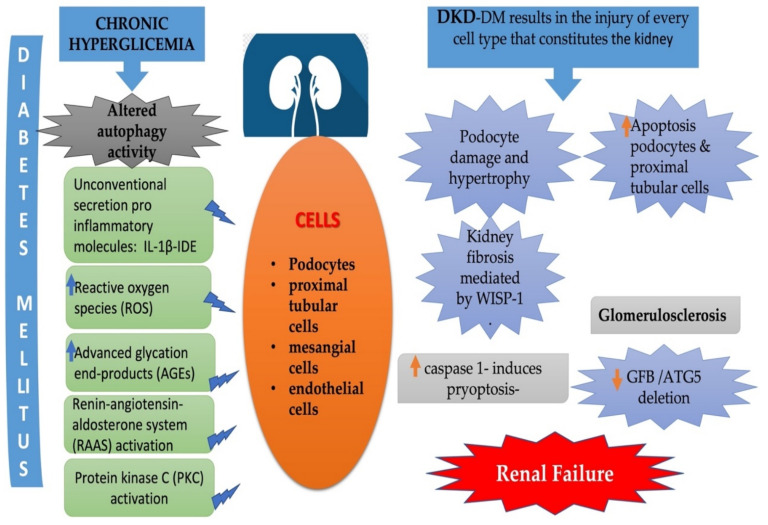
Altered Autophagy activity in DM results in the injury of every cell type that constitutes the kidney. Overview of structural changes in podocytes, proximal tubule epithelial cells, mesangial and endothelial cells.

As mentioned, impaired autophagy generates the accumulation of damaged organelles, such as mitochondria, which play a major role in the formation of reactive oxygen species (ROS). These also contribute to kidney damage, owing to the accumulation of impaired products that trigger apoptosis in renal cells, especially podocytes. In short, all the phenomena attributed to DM result in the injury of every cell type that constitutes the kidney. (Figure 2).

We describe the structural changes in podocytes and proximal tubule epithelial cells in Table 1.

### 3.1. Podocytes

The study of podocytes has piqued the interest of investigators because their damage can carry irreversible deleterious consequences to the kidney. Podocytes or glomerular epithelial cells constitute a critical component of the glomerular filtration barrier (GFB). As podocytes have no capacity to regenerate themselves, their loss, when significant, will subsequently result in an alteration of the GBF, leading to proteinuria and renal failure [63,64,65,66].

Under basal conditions, a high rate of autophagy maintains podocyte function. This autophagic flux is greater than that of any other kidney cell and is crucial for its subsistence [52]. Recent studies have shown that mTOR activation results in podocyte damage [54] and possibly contributes to podocyte hypertrophy [53]. Moreover, the deletion of Atg5, an autophagy regulator, results in autophagy inhibition, which causes glomerulosclerosis and damage to the GFB. These findings suggest that autophagy plays an important role in preserving the integrity of podocytes and contributes to the maintenance of normal kidney function [53].

A decrease in podocyte number represents one of the chief histological changes observed in DKD [67]. The injury of these cells could be a major clinical predictor of the progression of the disease [68,69] and is responsible for micro- and macroalbuminuria [70]. Several monogenic mutations associated with albuminuria in humans link specifically to some important proteins related to podocyte survival [71].

In animal studies, dysregulation of podocytes physiology is associated with the aggravation of albuminuria [72,73,74]. These models have estimated that a podocyte population loss beyond 20% is associated with irreversible glomerular damage and eventually, end-stage renal disease (ESRD) [75]. We can link several other histological alterations to irreversible kidney damage, including changes in basal membrane constitution. These alterations eventually impair podocyte adhesion molecules, generating a series of deleterious chain reactions [74].

Even when albuminuria is a marker of renal deterioration in diabetes, a considerable number of patients with DKD do not develop albuminuria [76,77]. Other DKD biomarkers that can even precede microalbuminuria development include podocyte-release products that are specific markers of the “health status” of these cells. Nephrin is a transmembrane protein involved in the regulation of the podocyte cytoskeleton. It is present in the urine of approximately 54% of patients without albuminuria and has shown to be an early DKD biomarker [78]. Reduction in the expression of this protein accompanies DM, resulting in alteration of the slit diaphragm complex by abnormal rearrangement of actin [79]. Hence, effacement of the foot process occurs, which decreases the integrity of podocyte cells, and contributes to an aberrant GFB. The action of DM is also involved in the dysregulation of other constituents responsible for actin cytoskeleton remodeling, such as the RHO family of small GTPases, RhoA, Cdc-42, and Rac1 [80]. For instance, the stimulation of Rho-Gtpase activity results in the alteration of cell motility and foot process effacement. In diabetic mice, rapamycin reduces albumin excretion, fusion of podocyte foot process, glomerular basement membrane thickening, and matrix accumulation [49]. By inhibiting mTOR, rapamycin increases LC3-expressing podocytes and autophagy, inducing the upregulation of nephrin in the glomeruli [49]. Diabetes also causes podocyte loss by increasing the expression of α3β1 integrin. This phenomenon prevents the adhesion of podocytes to the glomerular basement membrane; therefore, the resultant detachment between them disintegrates the entire structure [50,51]. Research has suggested that integrin-mediated cell attachment to the extracellular matrix modulates the autophagy response, thus influencing cell survival after a significant loss of cell-matrix contact [81]. In summary, autophagy appears to associate with several processes that regulate GFB integrity.

Oxidative stress plays a significant role in podocyte cell injury because it produces an imbalance in the TGF-β signaling pathway. This action thus alters autophagy and activates the inflammatory cascade, while increasing apoptosis [82]. As the autophagy-lysosome pathway is downregulated, a switch towards the ubiquitin-proteasome system tends to reduce damage. However, this process is not as efficient, resulting in the accumulation of impaired organelles and misfolded proteins. This series of events may result in podocyte death due to the activation of pro-apoptotic signals [52,53]. 

Finally, inflammation also predicts the loss of podocytes. Hyperglycemia activates pattern recognition receptors such as the nucleotide-binding domain, leucine-rich repeat, and pyrin domain-containing-3 (NLRP3), which activates the inflammasome and accelerates the development of DKD in diabetes patients. Inflammation is one of the main factors that contribute to podocyte damage because there is a high rate of caspase 1 that cleaves IL-1β, leading to pyroptosis, a highly inflammatory form of programmed cell death. Furthermore, some studies conducted in diabetic mice have shown that inactivating IL-1β decreases the progression of DKD through the reduction of pyroptosis [59,60]. As mentioned, autophagy is simultaneously involved in the pathophysiology of inflammation and the IL-1β secretion process. However, the clinical implications of this phenomenon remain unclear.

### 3.2. Proximal Tubule Epithelial Cells (PTEC)

Under basal conditions, PTEC generally has a lower level of autophagy in mice compared to that in podocytes [47]. However, active transport in tubular epithelial cells consumes large amounts of energy, making these cells more exposed to hypoxia or energy deprivation [59]. In addition, the renal medulla offers a more hypoxic environment than the renal cortex, compromising tubular epithelial cells substantially [60,83].

As mentioned previously, normal autophagy ensures cell vitality in hostile environments. However, this process is crucial for tubular cells; it has a more significant influence on PTEC than distal tubules and collecting ducts do. When autophagy fails in PTEC, the kidneys develop structural alterations, such as interstitial fibrosis. In addition, damaged mitochondria and alterations in cellular transport mechanisms contribute to severe alterations in renal homeostasis [47,59,84].

Chronic hyperglycemia generates impaired autophagy and increases the rate of senescence in PTECs [46,47]. Mouse models of diabetes show alteration of ATG genes, specifically the deletion of Atg5 and Atg7 in PTEC, which accompanies the accumulation of damaged mitochondria, tubular cell apoptosis, and fibrosis [61]. Other studies have shown that AGE-RAGE interaction lowers lysosomal activity, leading to the accumulation of abnormal molecules in PTEC, thereby decreasing cell survival. Albuminuria, frequently observed in diabetic patients, may trigger autophagy in PTECs in the short term. Excessive protein excretion alters autophagy when it becomes chronic, leading to progressive tubular injury [62]. However, mTOR mediates this effect. 

Transforming growth factor-beta 1 (TGF-β1) is one of the main regulators of kidney fibrosis [85] due to its capacity to act as an autophagy mediator. It promotes vacuole formation, LC3 expression, phosphorylation of P13K, and modulation of mTOR in human kidney cells [86]. It appears that WNT1-inducible signaling pathway protein-1 (WISP-1) might be involved in the evolution of kidney fibrosis through the process of autophagy too [56], especially in tubular epithelial cells. According to Wang, Chong, Shang, and Maiese [56], WISP-1 appears to decrease the expression of LC3 and BECN1 while increasing p62 activity in neuronal cell cultures. In rats, the increase or decrease in the expression of WISP-1 correlates with tissue response to the fibrotic stimuli prompted by TGF-B1. This suggests an existing relationship between them [87].

## 4. Diabetic Kidney Disease (DKD) and Autophagy

The duration of diabetes determines at least in part the difference between the two types of diabetes in terms of the prevalence of DKD. Other determinants include diverse associated comorbidities such as obesity, aging, insulin resistance, hypertension, and vascular diseases, including atherosclerosis [3].

The renal community regards DKD as the main cause of end-stage renal disease (ESRD) [20] in both developing and developed countries. Treatment of the end stages of the disease requires dialysis and/or transplantation. In many countries, DKD is present in at least 50% of patients requiring renal replacement treatment [88] and is responsible for the increased morbidity and mortality of patients [4]. For that matter, it places a huge financial burden on health insurance systems, especially considering the need for long-term replacement therapies [89]. There have been several identified risk factors that contribute to the progression of DKD, including hyperglycemia, hypertension, cardiovascular disease, obesity, and dyslipidemia. However, changes in lifestyle habits and/or pharmacological treatments can mitigate the effects of these conditions [55]. 

The Diabetes Complication and Treatment (DCCT) trial showed that intensive glycemic control could reduce the progression of DKD in T1DM [90]. In patients with T2DM, the United Kingdom Prospective Diabetes Study (UKDPS) trial showed that intensive blood glucose control resulted in a 33% reduction in the relative risk of development of microalbuminuria or clinical grade proteinuria after 12 years. The latter trial also showed a significant reduction in the percentage of patients doubling their plasma creatinine [91]. Similarly, the VADT study indicated that 6 years of intensive glycemic control resulted in marked reductions in renal outcomes after a 15-year follow-up [92]. In contrast, other studies have suggested that intensive glycemic control can reduce albuminuria and proteinuria but is insufficient to improve renal outcomes [91]. This necessitates a better understanding of the interplay between different factors, to elucidate the pure effect of hyperglycemia on the development and progression of DKD. 

When persistently elevated glycemia exceeds the capacity of body antioxidant defenses, it leads to an increase in reactive oxygen species (ROS) [1]. In addition, hyperglycemia contributes to the assembly of advanced glycation end-products (AGEs), activation of the renin-angiotensin-aldosterone system (RAAS), disruption of the klotho anti-aging factor, and the activation of protein kinase C (PKC) among other damaging processes. All these effects, including the downregulation of vitamin D receptors, have accompanied DKD [21].

Renal glucose excretion increases proportionally with increasing glycemia. Under physiological conditions, the glomeruli filter approximately 180g of glucose per day. The proximal tubules then reabsorb almost all of the filtered glucose. The glucose-sodium coupled transporter, SGLT2, within the proximal tubules, is responsible for reabsorbing 90% of the glucose filtered at the glomerulus. Another transporter, SGLT1, acts on the other 10% [93]. These homeostatic mechanisms change in patients with diabetes. Renal glucose resorption also increases in patients with diabetes. The pathophysiology of DKD encompasses the alterations in several coupled mechanisms linked with glucose renal resorption. These changes may directly and/or indirectly affect autophagy flux and rate in kidney tissues. In addition, alterations in autophagy may result in significant imbalances in renovascular physiology, with implications for the progression of DKD, as we will discuss below.

Inflammatory mechanisms play an important role in the pathophysiology of DKD. Dysregulation between pro- and anti-inflammatory mediators has a marked impact on several processes associated with DKD evolution. These mediators can affect autophagy in several ways. These alterations in autophagy can profoundly alter the pro-inflammatory tone in renal tissues [94]. Not only is canonical autophagy associated with the inflammatory status, but SA may be implicated in: (1) some inflammatory mediators, such as IL-1beta, released through this pathway [41]; (2) some inflammation-related enzymes in DKD, such as insulin-degrading enzyme (IDE), which are also released following an autophagy-linked secretion process; and (3) a close interplay between secretory and canonical autophagy [16].

Autophagy has been described as a benign mechanism that preserves renal function [21]. Under normal conditions, autophagy flux is critical for maintaining renal podocytes, proximal tubular epithelial cells, and mesangial and endothelial cell physiology, contributing to renal homeostasis. However, chronic hyperglycemia induces significant changes in the autophagic rate and flux, thus contributing to cell damage and progression of DKD [55]. It is worth considering these roles of autophagy in the kidneys when helping patients through the development of innovative therapeutic strategies to delay or prevent nephropathy [95].

## 5. Potential Implications for the Pharmacological Treatment and Prevention of DKD. Focus on New Antidiabetic Agents

In addition to their effects on blood glucose levels, some antidiabetic agents may modify canonical and non-canonical autophagy, adding potential benefits to DKD prevention and/or treatment (Table 1). Some of these agents activates AMPK and/or inhibit mTOR signaling (directly or indirectly). For some other, mechanisms are AMPK-mTOR independent (Figure 1).

Even when contraindicated in patients with low glomerular filtration rates, metformin (an antihyperglycemic agent widely used as first-line treatment for T2DM) has demonstrated significant effects on autophagy in several tissues and under different experimental conditions. It increases AMPK activity, which in turn inhibits mTOR [96]. Metformin may also inhibit mTOR independently of AMPK. Since mTOR inhibition leads to increased removal of autophagic material, research has suggested that metformin promotes the generation and subsequent elimination of autophagic vesicles through the inhibition of mTOR [87]. In a diabetic rat model, by high-fat feeding and an intraperitoneal injection of streptozotocin, metformin was associated with renoprotective effects by upregulating autophagy [97]. Metformin also reduced oxidative stress in renal tissue and correlated with reduced structural changes in the glomeruli. Metformin also inhibited the expression of the extracellular matrix. A recent study showed that Sirt1 inhibition partially blocked the protective effect of metformin on the kidney. This suggests that metformin may have some protective effects against kidney damage through induction of the Sirt1/FoxO1 pathway [97]. Moreover, in rat mesangial cells cultured under high glucose concentrations, metformin upregulated autophagy and reduced abnormal cell proliferation through the AMPK/SIRT1-FoxO1 pathway [98]. In animal models, metformin may protect the kidneys from chemical agents with proven nephrotoxic effects and is even more toxic than hyperglycemia. For instance, metformin appears to protect against cisplatin-induced toxicity by inducing autophagy via AMPK activation [99]. 

Some of the newest agents used in the treatment of T2DM have significant effects on autophagy. By inhibiting glucose reabsorption in the proximal tubules, SGLT2 inhibitors, such as empagliflozin, dapagliflozin, canagliflozin, and ertugliflozin, reduce blood glucose and body weight [100]. These agents have natriuretic effects and block Na/H^+^ exchanger NHE3, decreasing blood pressure, and modifying hemodynamics and endothelial function. In experimental models, these effects on the NHE3 exchanger modify intracellular and mitochondrial calcium concentrations, which may have significant physiological implications [101].

SGLT2 inhibitors decrease intraglomerular pressure. Under physiological conditions, these agents have marked effects on tubule-glomerular feedback, which maintain the glomerular filtration rate through modification of the preglomerular arteriole tone. Diabetes accompanies the increased expression of SGLT2 in the proximal tubules. This increases sodium and glucose reabsorption, thus reducing sodium concentration in the juxtaglomerular apparatus. Consequently, this affects the afferent arteriole tone and increases the intraglomerular pressure. This effect leads to hyperfiltration. An increased concentration of sodium delivery at the macula densa follows the SGLT2 inhibition, partially restoring the equilibrium in the glomerular vascular tone and intraglomerular pressure. These actions may explain, at least in part, the renoprotective effects that exhibited in both patients with and without type 2 diabetes [102]. Clinical trials carried out in patients with type 2 diabetes showed a consistent reduction in DKD progression in a statistically and clinically relevant manner [103]. 

Increased glucagon levels also accompany SGLT2 inhibition. Glucagon elevation is associated with lipolysis and ketogenesis. Hypothetically, ketone bodies could lead to more efficient energy generation in some tissues, such as the myocardium and renal tissues. The increased glucagon/insulin ratio complement AMPK activation and mTOR inhibition. These mechanisms drive an increased mitophagy rate. Research suggests that this effect facilitates mitochondrial physiology by restoring mitochondrial cycling and renovation [104]. However, the clinical relevance of these processes in humans remains unclear. Some of these effects may explain the reduction in the energy demands of the proximal tubule associated with SGLT inhibition, as observed in several experiments. The SGLT2 inhibitors increase erythropoietin levels via the hypoxia-inducible factor (HIF). This effect results in an increase in hematocrit, which may explain some of the clinical outcomes associated with the use of these agents in clinical practice [105].

Moreover, SGLT2 inhibitors have demonstrated the ability to reduce sympathetic nervous system activity [106]. Sympathetic stimulation increases tubular Na-K-ATPase activity, and sodium reabsorption and retention. In addition, activation of beta-1 adrenergic receptors in the juxtaglomerular apparatus are responsible for upregulating renin release, with a general increase in the renin-angiotensin-aldosterone system (RAAS). Overactivation of the RAAS plays a critical role in DKD progression; RAAS blockade plays an important role in the prevention and treatment of DKD [107]. By reducing sympathetic system and renal activities, SGLT2 inhibitors induce several beneficial hemodynamic and metabolic changes. This contributes to a reduction in oxidative stress and inflammation. 

In sum, these effects on glycemia, tubule-glomerular feedback, renal hemodynamics, energy generation, oxygen consumption, and oxidative stress may have important implications on the inflammatory processes that usually characterize some phases of DKD [108].

Many of the effects of SGLT2i may result in important changes in the autophagy rate and flux, as well as in SA. Glucagon increases, for instance, induces autophagy in several tissues [109]. In contrast, SGLT1 inhibitors have proven to directly stimulate autophagy in cardiomyocytes through AMPK, sirtuin-1, and hypoxia-inducible factors-1α/2α [110]. In db/db rodents, empagliflozin demonstrably enhanced the areas of glomerular staining for beclin-1 and LAMP-1, two widely used markers of autophagy [111]. The volume density of autophagosomes and autolysosomes in podocytes increased. These effects may increase podocyte survival and protect against GFB. Research suggests that SGLT2 inhibitors activate SIRT1/AMPK, suppress Akt/mTOR signaling and modulate autophagy [112]. As a result, it restores mitochondrial function, reducing oxidative stress and inflammation [113]. We cannot dismiss the potential additional effects of these agents on SA. However, there is yet a poor understanding of the direct and indirect effects of SGLT2i on SA and this merits further research.

GLP1 receptor agonists (exenatide, liraglutide, dulaglutide, semaglutide, etc.) are highly effective antidiabetic agents with some renoprotective effects mediated by GLP-1 receptor signaling [114]. They have shown some natriuretic effects in rats [115]. These GLP1 receptor agonists decrease proximal sodium reabsorption by reducing NHE3 transport activity and research suggests that they may increase the glomerular filtration rate [116]. Reportedly, liraglutide suppresses autophagy in human kidney-2 cells and diabetic rat kidneys [117]. In experimental models, however, GLP-1 appears to regulate autophagy flux positively through the AMPK-mTOR signaling pathway [117,118,119,120]. In addition, GLP1 receptor agonists can also contribute to the restoration of autophagy balance in kidney tissues through the reduction of glycemia, inflammation, and oxidative stress [118,119].

Other non-antidiabetic nephroprotective agents induce profound changes in autophagy. Renin-angiotensin-aldosterone system inhibitors (RAASi) and mineralocorticoid receptor antagonists frequently used in clinical practice are among them [120]. As with metformin, SGLT2 inhibitors and GLP1 agonists these agentes modulate AMPK-mTOR signalling but also exhibit mTOR independent mechanisms of action. Other new agents under research appear to modify autophagy rates and flux by AMPK-mTOR dependent and independent mechanisms: bardoxolone (an activator of antioxidant pathways that acts on nuclear factor-erythroid 2-related factor 2 [Nrf2]), apoptosis signal-regulating kinase (ASK)-1 inhibitors, and several drugs with anti-inflammatory effects are among them [120]. Unmet needs and potential gaps in that area merit further research. Autophagy and drugs in DKD are summarized in Table 2.

As mentioned, some of these compounds have certain mechanisms in common. Rapamycyn, everolimus and other mTORC1 inhibitors have well recognized effects in vitro as well as in vivo. Rapamycin was initially discovered as an antifungal agent and possess immunosuppressive and anti-proliferative effects in eukariotic cells. In renal cells, rapamycin augments autophagy by inhibiting the mTORC1-ULK1 pathway. In MRLlpr/lprmice model of Lupus Nephritis rapamycin induces autophagy upregulation through mTORC1 inhibition [121,122]. In this model, rapamycin-associated increase in autophagy resulted cytoprotective against podocyte injury by antibody and interferon [122]- Other agents increases kidney autophagy rate in animals by mTOC1-ULK1 signaling inhibition: ursolic acid, notoginsenoside R1, pyridoxal-derivates among them [122]. Astragaloside upregulates autophagy in kidneys of streptozotocin-induced diabetic animals via AMPK-mTORC1. In transplant biopsies of human patients receiving rapamycin, electron microscopy showed a significant increase in podocyte autophagosomal volume fractions when compared with patients treated without mTOR inhibitors [123]. However, more research is needed to understand the potential functional consequences of this observation on clinical outcomes in patients with DKD treated with mTOR inhibitors [123], and many agents have been associated with an AMPK-mTOR or mTOR-ULK independent increase in autophagy in renal cells (carbamazepine, minoxidil, xetospongin B, atrasentan, for instance) [121] (Figure 1). Even when several agents have common mechanisms of action that may explain some beneficial effects on DKD progression, it is important to point out that almost all the agents mentioned in this description have multiple effects on several non-autophagy related mechanisms with potential clinical benefits. The specific role of autophagy modulation on DKD evolution is still uncertain and deserves for further investigations. 

## 6. Some Areas of Uncertainty and Suggestions for Future Research

Relevant information on the role of autophagy in the pathophysiology of DKD is still missing. Many of the published studies do not explore autophagy flux and remain confined to a static description of the process. Canonical pathways are more frequently described than other non-classical processes, including secretory autophagy. Besides, studies on the interlinks between macroautophagy and secretory autophagy are lacking in the literature.

As mentioned before, the precise role of autophagy on the renal effects of drugs with potential benefits on DKD outcomes remains as one of the most relevant gaps in knowledge. Complexity of metabolic, hemodynamic, neural, cellular, subcellular and microenvironmental events are difficult to disect to evaluate the specfic role of autophagy modulation on outcomes. In addition, many of these actions were observed and described in ‘in vitro’ and/or in animal models of the disease. Uncertainty remains high on the extrapolation of these effects to human beings. On the other hand, evaluation of autophagy in living human kidney tissues is difficult. Well powered studies on series of biopsies from patients at different stages of evolution, with different comorbidities, complications, ages and degrees of metabolic control are almost impossible on rutinary basis. DKD represents a very heterogeneous population with a high load of patient individual covariates; as a result, this kind of studies would require a considerable number of patients to keep imprecision under control. In general, well controlled dose-response studies in human are also lacking.

As observed by Tang and col. [21] some of the effects of any specific agent may result beneficial for some processes and detrimental for other in the same tissue. In addition, autophagy may play different roles in different cell types. In summary, critical information on modification of autophagy by potentially nephroprotective agents in different DKD stages and conditions is still lacking. The contribution of autophagy modulators on DKD outcomes in humans remains unclear and deserves for further research.

## 7. Conclusions

Impaired autophagy is involved in the pathophysiology of DKD, which increases ROS formation, induces kidney cell damage and apoptosis, and mediates inflammatory responses and fibrosis. Glomeruli, tubules, interstitial tissues, and the vascular renal compartment suffer from the impact of autophagy dysregulation observed in several stages of chronic kidney disease (CKD). At the same time, alterations in the renal microstructure and physiology promote relevant changes in the surrounding environment. These, in turn, induce new modifications in autophagy rates and flux. Several factors contribute to the impact of autophagy dysregulation: age, diabetes duration, metabolic control (including glycemic and lipid levels), inflammatory factors, and hypertension. Genetic and epigenetic factors are also influential factors. Diabetes is the primary cause of CKD in the occidental world and, ultimately, this highly prevalent complication leads to increased mortality, morbidity, and deterioration of quality of life. However, some new antidiabetic drugs appear to exhibit renoprotective effects. These agents seem to modify autophagy significantly through several mechanisms. Other potentially nephroprotective non-antidiabetic drugs appear to partially reverse autophagy dysregulation. The modulation of autophagy could be relevant for the pharmacological treatment and prevention of DKD in the future. Therefore, this is an area that requires further research.

## Figures and Tables

**Table 1:** Autophagy in kidney physiology and DKD pathophysiology.

Autophagy in Kidney Physiology	Impaired Autophagy in DKD
Essential for kidney’s integrity and proper functioning [47]. Protective role over the kidneys by preventing fibrosis [48]. Protective role preventing inflammation [43]. Regulates GFB integrity [48,49,50,51]. Contributes to renal homeostasis by regulating autophagy flux in kidney cells [52]. May increase glucagon level, which induces autophagy in several tissues.	Generates the accumulation of impaired organelles and misfolded proteins: The accumulation of mitochondria plays a huge part in the formation of reactive oxygen species (ROS) [1]. This activates pro apoptotic signals and may result in podocyte death. [52,53] Induces podocyte damage and hypertrophy [54,53]. Induces apoptosis in podocytes and proximal tubular cells [55]. Mediator of unconventional secretion of pro-inflammatory molecules such as IL-1β [30] and IDE [45]. Develops kidney fibrosis mediated by WISP-1 [56]. Set in motion glomerulosclerosis and damage in the GFB by the deletion of Atg5 [53]. Increases the rate of senescence in PTEC [57,58]. Up-regulates nephrin in the glomeruli through inhibition of mTOR, altering podocytes cytoskeleton [49]. Induces pryoptosis, a highly inflammatory form of programmed cell death due to a high rate of caspase 1 that cleaves IL-1B [59,60]. Produces alterations in ATG genes leading to kidney damage [61]. Inhibition of mTOR may increase protein excretion, which promotes progressive tubular injury [62].

**Table 2:** Autophagy and drugs in DKD.

Agent	Key Mechanisms	Additional Comments
Metformin [99,100]	Modifies autophagy by activating AMPK and inhibiting mTOR. Induction of Sirt1/FoxO1.	First-line treatment in patients with type 2 DM.
SGLT2 inhibitors (e.g., canagliflozin, dapagliflozin, empagliflozin, other) [100,101,102,103,104,105,106,107,108,109,110,111,112,113]	It would activate SIRT1/AMPK, suppress Akt/mTOR signaling. Multiple indirect effects (associated with changes in glucagon, HIF and EPO concentrations hemodynamic effects, changes in glomerulo-tubular balance, in RAAS, sympathetic tone, etc.).	Relevant benefits on renal preservation consistently proven in human beings in several clinical trials. Proven cardiovascular benefits, particularly in patients with heart failure.
GLP1 receptor agonists (liraglutide, semaglutide, dulaglutide, other) [114,115,116,117,118]	GLP1 receptor mediated actions: AMPK-dependent and -independent mechanisms (natriuresis, effects on NH3, other).	Some positive effects observed in clinical trials. Proven cardiovascular benefits. Role of autophagy as a potential explanation for these benefits still uncertain.
Renin-angiotensin-aldosterone system inhibitors (RAASi) and mineralocorticoid receptor antagonists [120,121]	Direct (AMPK- and mTOR-mediated) and indirect mechanisms associated with reduction in RAAS activity.	Clinically proven benefits on renal preservation. Role of autophagy as a potential explanation for these benefits still uncertain.
Rapamycin, everolimus, other mTOR inhibitors [121,122,123]	Direct mTOR inhibition	Autophagy upregulation in several models and in biopsies of human transplants.
Other (investigational) agents [120]	Factor-erythroid 2-related factor 2 [Nrf2] activators. Apoptosis signal-regulating kinase (ASK)-1 inhibitors. Several drugs with anti-inflammatory properties.	Clinical benefits on renal outcomes still to be demonstrated.

## Data Availability

Not applicable.

## Abbreviations/Acronyms

ULK1	unc-51-like kinase 1
VMP1	Vacuole membrane protein 1
PI3KC3	Phosphatidylinositol 3-kinase C3
FYVE	FYVE zinc finger domain
DFCP1	Double FYVE containing protein 1
PI3P	Phosphatidylinositol 3-phosphate
GLP1	Glucagon like peptide 1
ATG	Autophagy-related gen or protein: ATG12-ATG5 complex
LC3	Microtubule-associated proteins 1A/1B light chain 3B (also known as MAP1LC3B)
LAMP	Lysosomal-associated membrane protein
WIPI	WD repeat domain phosphoinositide-interacting protein
ATG12-ATG5-ATG16	complex
STX17	Sintaxin 17
RAB7	Ras-related protein 7
mTOR	mammalian Target of Rapamycin
SGLT2	Sodium Glucose Cotransporter 2
AMPK	AMP-activated Kinase
BECLIN 1	mammalian ortholog of the yeast autophagy-related gene 6 (Atg6) and BEC-1 in the C. elegans nematode
BECN1	Coiled-Coil Moesin-Like BCL2-Interacting Protein
Vps34	PI3K activity of the catalytic subunit

## References

[1] González C., Lee M., Marchetti P., Pietropaolo M., Towns R., Vaccaro M.I., Watada H., Wiley J.W. (2011). The emerging role of autophagy in the pathophysiology of diabetes mellitus. Autophagy.

[2] Saeedi P., Petersohn I., Salpea P., Malanda B., Karuranga S., Unwin N., Colagiuri S., Guariguata L., Motala A.A., Ogurtsova K. (2019). Global and regional diabetes prevalence estimates for 2019 and projections for 2030 and 2045: Results from the International Diabetes Federation Diabetes Atlas, 9th edition. Diabetes Res. Clin. Pract..

[3] Thomas M., Brownlee M., Susztak K., Sharma K., Jandeleit-Dahm K.A.M., Zoungas S., Rossing P., Groop P.H., Cooper M.E. (2015). Diabetic kidney disease. Nat. Rev. Dis. Primers.

[4] Ding Y., Choi M. (2014). Autophagy in diabetic nephropathy. J. Endocrinol..

[5] Mortimore G., Poso A. (1987). Intracellular Protein Catabolism and its Control during Nutrient Deprivation and Supply. Annu. Rev. Nutr..

[6] Yamano K., Fogel A., Wang C., van der Bliek A., Youle R. (2014). Mitochondrial Rab GAPs govern autophagosome biogenesis during mitophagy. Elife.

[7] Lee J., Giordano S., Zhang J. (2011). Autophagy, mitochondria and oxidative stress: Cross-talk and redox signalling. Biochem. J..

[8] Kaarniranta K., Sinha D., Blasiak J., Blasiak J., Kauppinen A., Veréb Z., Salminen A., Boulton M.E., Petrovski G. (2013). Autophagy and heterophagy dysregulation leads to retinal pigment epithelium dysfunction and development of age-related macular degeneration. Autophagy.

[9] Cuervo A., Wong E. (2013). Chaperone-mediated autophagy: Roles in disease and aging. Cell Res..

[10] Choi A.M., Ryter S.W., Levine B. (2013). Autophagy in human health and disease. N. Engl. J. Med..

[11] Zahoor M., Farhan H. (2018). Crosstalk of Autophagy and the Secretory Pathway and Its Role in Diseases. Int. Rev. Cell Mol. Biol..

[12] Packer M. (2020). Role of Impaired Nutrient and Oxygen Deprivation Signaling and Deficient Autophagic Flux in Diabetic CKD Development: Implications for Understanding the Effects of Sodium-Glucose Cotransporter 2-Inhibitors. J. Am. Soc. Nephrol..

[13] Klionsky D., Abdalla F., Abeliovich H., Abraham R.T., Acevedo-Arozena A., Adeli K., Agholme L., Agnello M., Agostinis P., Aguirre-Ghiso J.A. (2012). Guidelines for the use and interpretation of assays for monitoring autophagy. Autophagy.

[14] Ponpuak M., Mandell M., Kimura T., Chauhan S., Cleyrat C., Deretic V. (2015). Secretory autophagy. Curr. Opin. Cell Biol..

[15] Sudhof T., Rizo J. (2011). Synaptic Vesicle Exocytosis. Cold Spring Harb. Perspect. Biol..

[16] González C., Resnik R., Vaccaro M. (2020). Secretory Autophagy and Its Relevance in Metabolic and Degenerative Disease. Front. Endocrinol..

[17] Rabouille C., Malhotra V., Nickel W. (2012). Diversity in unconventional protein secretion. J. Cell Sci..

[18] Jiang S., Dupont N., Castillo E., Deretic V. (2013). Secretory versus Degradative Autophagy: Unconventional Secretion of Inflammatory Mediators. J. Innate Immun..

[19] New J., Thomas S. (2019). Autophagy-dependent secretion: Mechanism, factors secreted, and disease implications. Autophagy.

[20] Grasso D., Renna F., Vaccaro M. (2018). Initial Steps in Mammalian Autophagosome Biogenesis. Front. Cell Dev. Biol..

[21] Tang C., Livingston M.J., Liu Z., Dong Z. (2020). Autophagy in kidney homeostasis and disease. Nat. Rev. Nephrol..

[22] Lee J., Park S., Takahashi Y., Wang H. (2010). The Association of AMPK with ULK1 Regulates Autophagy. PLoS ONE..

[23] Inoki K., Zhu T., Guan K. (2003). TSC2 Mediates Cellular Energy Response to Control Cell Growth and Survival. Cell.

[24] Gwinn D.M., Shackelford D.B., Egan D.F., Mihaylova M.M., Mery A., Vasquez D.S., Turk B.E., Shaw R.J. (2008). AMPK phosphorylation of raptor mediates a metabolic checkpoint. Mol. Cell.

[25] Dusetti N., Jiang Y., Vaccaro M., Tomasini R., Azizi Samir A., Calvo E.L., Ropolo A., Fiedler F., Mallo G.V., Dagorn J.-C. (2002). Cloning and Expression of the Rat Vacuole Membrane Protein 1 (VMP1), a New Gene Activated in Pancreas with Acute Pancreatitis, Which Promotes Vacuole Formation. Biochem. Biophys. Res. Commun..

[26] Ropolo A., Grasso D., Pardo R., Sacchetti M.L., Archange C., Lo Re A., Seux M., Nowak J., Gonzalez C.D., Iovanna J.L. (2007). The Pancreatitis-induced Vacuole Membrane Protein 1 Triggers Autophagy in Mammalian Cells. J. Biol. Chem..

[27] Nascimbeni A., Giordano F., Dupont N., Grasso D., Vaccaro M.I., Codogno P., Morel E. (2017). ER–plasma membrane contact sites contribute to autophagosome biogenesis by regulation of local PI 3P synthesis. EMBO J..

[28] Mizushima N. (2020). The ATG conjugation systems in autophagy. Curr. Opin. Cell Biol..

[29] Rogov V., Dötsch V., Johansen T., Kirkin V. (2014). Interactions between Autophagy Receptors and Ubiquitin-like Proteins Form the Molecular Basis for Selective Autophagy. Mol. Cell..

[30] Nanao T., Koike M., Yamaguchi J., Sasaki M., Uchiyama Y. (2015). Cellular localization and tissue distribution of endogenous DFCP1 protein. Biomed. Res..

[31] Wilson M., Dooley H., Tooze S. (2014). WIPI2b and Atg16L1: Setting the stage for autophagosome formation. Biochem. Soc. Trans..

[32] Otomo C., Metlagel Z., Takaesu G., Otomo T. (2012). Structure of the human ATG12~ATG5 conjugate required for LC3 lipidation in autophagy. Nat. Struct. Mol. Biol..

[33] Nakatogawa H., Ichimura Y., Ohsumi Y. (2007). Atg8, a Ubiquitin-like Protein Required for Autophagosome Formation, Mediates Membrane Tethering and Hemifusion. Cell.

[34] Lee Y., Lee J. (2016). Role of the mammalian ATG8/LC3 family in autophagy: Differential and compensatory roles in the spatiotemporal regulation of autophagy. BMB Rep..

[35] Ktistakis N., Tooze S. (2016). Digesting the Expanding Mechanisms of Autophagy. Trends Cell Biol..

[36] Noda N., Fujioka Y., Hanada T., Ohsumi Y., Inagaki F. (2012). Structure of the Atg12–Atg5 conjugate reveals a platform for stimulating Atg8–PE conjugation. EMBO Rep..

[37] Jiang P., Nishimura T., Sakamaki Y., Itakura E., Hatta T., Natsume T., Mizushima N. (2014). The HOPS complex mediates autophagosome–lysosome fusion through interaction with syntaxin 17. Mol. Biol. Cell.

[38] Gutierrez M. (2004). Rab7 is required for the normal progression of the autophagic pathway in mammalian cells. J. Cell Sci..

[39] Diao J., Liu R., Rong Y., Zhao M., Zhang J., Lai Y., Zhou Q., Wilz L.M., Li J., Vivona S. (2015). ATG14 promotes membrane tethering and fusion of autophagosomes to endolysosomes. Nature.

[40] Xu J., Camfield R., Gorski S. (2018). The interplay between exosomes and autophagy–partners in crime. J. Cell Sci..

[41] Nilsson P., Loganathan K., Sekiguchi M., Matsuba Y., Hui K., Tsubuki S., Tanaka M., Iwata N., Saito T., Saido T.C. (2013). Aβ Secretion and Plaque Formation Depend on Autophagy. Cell Rep..

[42] Kimura T., Jia J., Kumar S., Choi S.W., Gu Y., Mudd M., Dupont N., Jiang S., Peters R., Farzam F. (2016). Dedicated SNARE s and specialized TRIM cargo receptors mediate secretory autophagy. EMBO J..

[43] Colombo M., Raposo G., Théry C. (2014). Biogenesis, Secretion, and Intercellular Interactions of Exosomes and Other Extracellular Vesicles. Annu. Rev. Cell Dev. Biol..

[44] Guo H., Chitiprolu M., Roncevic L., Javalet C., Hemming F.J., Trung M.T., Meng L., Latreille E., Tanese de Souza C., McCulloch D. (2017). Atg5 Disassociates the V1V0-ATPase to Promote Exosome Production and Tumor Metastasis Independent of Canonical Macroautophagy. Dev. Cell..

[45] Farris W., Mansourian S., Chang Y. (2003). Insulin-degrading enzyme regulates the levels of insulin, amyloid -protein, and the-amyloid precursor protein intracellular domain in vivo. Proc. Natl. Acad. Sci. USA.

[46] Radulescu R.T. (2008). Tumor suppressor and anti-inflammatory protein: An expanded view on insulin-degrading enzyme (IDE). arXiv.

[47] Liu S., Hartleben B., Kretz O., Wiech T., Igarashi P., Mizushima N., Walz G., Hu T.B. (2012). Autophagy plays a critical role in kidney tubule maintenance, aging and ischemia-reperfusion injury. Autophagy.

[48] De Almeida Barbosa A., Zhou H., Hültenschmidt D., Totovic V., Jurilj N., Pfeifer U. (1992). Inhibition of cellular autophagy in proximal tubular cells of the kidney in streptozotocin-diabetic and uninephrectomized rats. Virchows Arch. B Cell Pathol. Incl. Mol. Pathol..

[49] Xiao T., Guan X., Nie L., Wang S., Sun L., He T., Huang Y., Zhang J., Yang K., Wang J. (2014). Rapamycin promotes podocyte autophagy and ameliorates renal injury in diabetic mice. Mol. Cell Biochem..

[50] Chen H.C., Chen C.A., Guh J.Y., Chang J.M., Shin S.J., Lai Y.H. (2000). Altering expression of alpha3beta1 integrin on podocytes of human and rats with diabetes. Life Sci..

[51] Mathew S., Chen X., Pozzi A., Zent R. (2012). Integrins in renal development. Pediatr. Nephrol..

[52] Hartleben B., Gödel M., Meyer-Schwesinger C., Liu S., Ulrich T., Köbler S., Wiech T., Grahammer F., Arnold S.J., Lindenmeyer M.T. (2010). Autophagy influences glomerular disease susceptibility and maintains podocyte homeostasis in aging mice. J. Clin. Investig..

[53] Lenoir O., Jasiek M., Hénique C., Guyonnet L., Hartleben B., Bork T., Chipont A., Flosseau K., Bensaada I., Schmitt A. (2015). Endothelial cell and podocyte autophagy synergistically protect from diabetes-induced glomerulosclerosis. Autophagy.

[54] Zschiedrich S., Bork T., Liang W., Wanner N., Eulenbruch K., Munder S., Hartleben B., Kretz O., Gerber S., Simons M. (2017). Targeting mTOR Signaling Can Prevent the Progression of FSGS. J. Am. Soc. Nephrol..

[55] Koch E., Nakhoul R., Nakhoul F., Nakhoul N. (2020). Autophagy in diabetic nephropathy: A review. Int. Urol. Nephrol..

[56] Yang X., Wang H., Tu Y., Li Y., Zou Y., Li G., Li W., Zhong X. (2019). WNT1-inducible signaling protein-1 mediates TGF-β1-induced renal fibrosis in tubular epithelial cells and unilateral ureteral obstruction mouse models via autophagy. J. Cell Physiol..

[57] Zhan M., Usman I.M., Sun L., Kanwar Y.S. (2015). Disruption of renal tubular mitochondrial quality control by Myo-inositol oxygenase in diabetic kidney disease. J. Am. Soc. Nephrol..

[58] Ma Z., Li L., Livingston M., Zhang D., Mi Q., Zhang M., Ding H.F., Huo Y., Mei C., Dong Z. (2020). p53/microRNA-214/ULK1 axis impairs renal tubular autophagy in diabetic kidney disease. J. Clin. Investig..

[59] Shahzad K., Bock F., Dong W., Shahzad K., Bock F., Dong W., Wang H., Kopf S., Kohli S., Al-Dabet M.M. (2015). Nlrp3-inflammasome activation in non-myeloid-derived cells aggravates diabetic nephropathy. Kidney Int..

[60] Tschopp J., Schroder K. (2010). NLRP3 inflammasome activation: The convergence of multiple signalling pathways on ROS production?. Nat. Rev. Immunol..

[61] Kimura T., Takabatake Y., Takahashi A., Kaimori J.Y., Matsui I., Namba T., Kitamura H., Niimura F., Matsusaka T., Soga T. (2011). Autophagy protects the proximal tubule from degeneration and acute ischemic injury. J. Am. Soc. Nephrol..

[62] Nolin A.C., Mulhern R.M., Panchenko M.V., Pisarek-Horowitz A., Wang Z., Shirihai O., Borkan S.C., Havasi A. (2016). Proteinuria causes dysfunctional autophagy in the proximal tubule. Am. J. Physiol. Ren. Physiol..

[63] Zeng C., Fan Y., Wu J., Shi S., Chen Z., Zhong Y., Zhang C., Zen K., Liu Z. (2014). Podocyte autophagic activity plays a protective role in renal injury and delays the progression of podocytopathies. J. Pathol..

[64] Wiggins R. (2007). The spectrum of podocytopathies: A unifying view of glomerular diseases. Kidney Int..

[65] He P., Liu D., Zhang B., Zhou G., Su X., Wang Y., Wang X., Li D. (2017). Hepatitis B Virus X Protein Reduces Podocyte Adhesion via Downregulation of α3β1 Integrin. Cell. Physiol. Biochem..

[66] Lin X., Zhen X., Huang H., Lin X., Zhen X., Huang H., Wu H., You Y., Guo P., Gu X. (2017). Role of MiR-155 Signal Pathway in Regulating Podocyte Injury Induced by TGF-β1. Cell. Physiol. Biochem..

[67] Siu B., Saha J., Smoyer W., Sullivan K., Brosius F. (2006). Reduction in podocyte density as a pathologic feature in early diabetic nephropathy in rodents: Prevention by lipoic acid treatment. BMC Nephrol..

[68] Meyer T.W., Bennett P.H., Nelson R.G. (1999). Podocyte number predicts long-term urinary albumin excretion in Pima Indians with Type II diabetes and microalbuminuria. Diabetologia.

[69] Pagtalunan M., Miller P., Jumping-Eagle S., Nelson R.G., Myers B.D., Rennke H.G., Coplon N.S., Sun L., Meyer T.W. (1997). Podocyte loss and progressive glomerular injury in type II diabetes. J. Clin. Investig..

[70] Jin J., Wu D., Zhao L., Zou W., Shen W., Tu Q., He Q. (2018). Effect of autophagy and stromal interaction molecule 1 on podocyte epithelial-mesenchymal transition in diabetic nephropathy. Int. J. Clin. Exp. Pathol..

[71] D’Agati V., Kaskel F.J., Falk R.J. (2011). Focal Segmental Glomerulosclerosis. N. Engl. J. Med..

[72] Wang W., Wang Y., Long J., Wang J., Haudek S.B., Overbeek P., Chang B.H., Schumacker P.T., Danesh F.R. (2012). Mitochondrial fission triggered by hyperglycemia is mediated by ROCK1 activation in podocytes and endothelial cells. Cell Metab..

[73] Niranjan T., Bielesz B., Gruenwald A., Ponda M.P., Kopp J.B., Thomas D.B., Susztak K. (2008). The Notch pathway in podocytes plays a role in the development of glomerular disease. Nat. Med..

[74] Kato H., Gruenwald A., Suh J.H., Miner J.H., Barisoni-Thomas L., Taketo M.M., Faul C., Millar S.E., Holzman L.B., Susztak K. (2011). Wnt/β-catenin pathway in podocytes integrates cell adhesion, differentiation, and survival. J. Biol. Chem..

[75] Rutkowski J.M., Wang Z.V., Park A.S., Zhang J., Zhang D., Hu M.C., Moe O.W., Susztak K., Scherer P.E. (2013). Adiponectin promotes functional recovery after podocyte ablation. J. Am. Soc. Nephrol..

[76] Kramer H.J., Nguyen Q.D., Curhan G., Hsu C.Y. (2003). Renal insufficiency in the absence of albuminuria and retinopathy among adults with type 2 diabetes mellitus. JAMA.

[77] Dahlquist G., Stattin E., Rudberg S. (2001). Urinary albumin excretion rate and glomerular filtration rate in the prediction of diabetic nephropathy; a long-term follow-up study of childhood onset type-1 diabetic patients. Nephrol. Dial. Transplant..

[78] Kravets I., Mallipattu S. (2020). The Role of Podocytes and Podocyte-Associated Biomarkers in Diagnosis and Treatment of Diabetic Kidney Disease. J. Endocr. Soc..

[79] Doublier S., Salvidio G., Lupia E., Ruotsalainen V., Verzola D., Deferrari G., Camussi G. (2003). Nephrin Expression Is Reduced in Human Diabetic Nephropathy: Evidence for a Distinct Role for Glycated Albumin and Angiotensin II. Diabetes.

[80] Peng F., Wu D., Gao B., Ingram A.J., Zhang B., Chorneyko K., McKenzie R., Krepinsky J.C. (2008). RhoA/Rho-Kinase Contribute to the Pathogenesis of Diabetic Renal Disease. Diabetes.

[81] Vlahakis A., Debnath J. (2017). The Interconnections between Autophagy and Integrin-Mediated Cell Adhesion. J. Mol. Biol..

[82] Lin J.S., Susztak K. (2016). Podocytes: The Weakest Link in Diabetic Kidney Disease?. Curr. Diab. Rep..

[83] Lin F. (2017). Autophagy in renal tubular injury and repair. Acta Physiol..

[84] Layton A. (2016). Recent advances in renal hypoxia: Insights from bench experiments and computer simulations. Am. J. Physiol. Ren. Physiol..

[85] Tanaka S., Tanaka T., Nangaku M. (2014). Hypoxia as a key player in the AKI-to-CKD transition. Am. J. Physiol. Ren. Physiol..

[86] Havasi A., Dong Z. (2016). Autophagy and Tubular Cell Death in the Kidney. Semin. Nephrol..

[87] Kim Y.C., Guan K.L. (2015). mTOR: A pharmacologic target for autophagy regulation. J. Clin. Investig..

[88] Collins A.J., Foley R.N., Chavers B., Gilbertson D., Herzog C., Ishani A., Johansen K., Kasiske B.L., Kutner N., Liu J. (2014). US Renal Data System 2013 Annual Data Report. Am. J. Kidney Dis..

[89] Saran R., Robinson B., Abbott K.C., Agodoa L.Y.C., Bhave N., Bragg-Gresham J., Balkrishnan R., Dietrich X., Eckard A., Eggers P.W. (2018). US Renal Data System 2017 Annual Data Report: Epidemiology of Kidney Disease in the United States. Am. J. Kidney Dis..

[90] Reidy K., Kang H., Hostetter T., Susztak K. (2014). Molecular mechanisms of diabetic kidney disease. J. Clin. Investig..

[91] Coca S.G., Ismail-Beigi F., Haq N., Krumholz H.M., Parikh C.R. (2012). Role of intensive glucose control in development of renal end points in type 2 diabetes mellitus: Systematic review and meta-analysis intensive glucose control in type 2 diabetes. Arch. Intern. Med..

[92] Agrawal L., Azad N., Bahn G., Reaven P.D., Hayward R.A., Reda D.J., Emanuele N.D. (2019). Intensive Glycemic Control Improves Long-term Renal Outcomes in Type 2 Diabetes in the Veterans Affairs Diabetes Trial (VADT). Diabetes Care.

[93] Rieg T., Vallon V. (2018). Development of SGLT1 and SGLT2 inhibitors. Diabetologia..

[94] Kimura T., Isaka Y., Yoshimori T. (2017). Autophagy and kidney inflammation. Autophagy.

[95] Sakai S., Yamamoto T., Takabatake Y., Takahashi A., Namba-Hamano T., Minami S., Fujimura R., Yonishi H., Matsuda J., Hesaka A. (2019). Proximal Tubule Autophagy Differs in Type 1 and 2 Diabetes. J. Am. Soc. Nephrol..

[96] Rena G., Hardie D.G., Pearson E.R. (2017). The mechanisms of action of metformin. Diabetologia.

[97] Xu J., Liu L., Xu L., Xing Y., Ye S. (2020). Metformin alleviates renal injury in diabetic rats by inducing Sirt1/FoxO1 autophagic signal axis. Clin. Exp. Pharmacol. Physiol..

[98] Ren H., Shao Y., Wu C., Ma X., Lv C., Wang Q. (2020). Metformin alleviates oxidative stress and enhances autophagy in diabetic kidney disease via AMPK/SIRT1-FoxO1 pathway. Mol. Cell Endocrinol..

[99] Li J., Gui Y., Ren J., Liu X., Feng Y., Zeng Z., He W., Yang J., Dai C. (2016). Metformin Protects against Cisplatin-Induced Tubular Cell Apoptosis and Acute Kidney Injury via AMPKα-regulated Autophagy Induction. Sci. Rep..

[100] Mima A. (2018). Renal protection by sodium-glucose cotransporter 2 inhibitors and its underlying mechanisms in diabetic kidney disease. J. Diabetes Complicat..

[101] Edwards A., Auberson M., Ramakrishnan S.K., Bonny O. (2019). A model of uric acid transport in the rat proximal tubule. Am. J. Physiol. Ren. Physiol..

[102] Neuen B.L., Young T., Heerspink H.J.L., Neal B., Perkovic V., Billot L., Mahaffey K.W., Charytan D.M., Wheeler D.C., Arnott C. (2019). SGLT2 inhibitors for the prevention of kidney failure in patients with type 2 diabetes: A systematic review and meta-analysis. Lancet Diabetes Endocrinol..

[103] Kashihara N., Kidokoro K., Kanda E. (2020). Renoprotective effects of sodium-glucose cotransporter-2 inhibitors and underlying mechanisms. Curr. Opin. Nephrol. Hypertens..

[104] Esterline R., Vaag A., Oscarsson J., Vora J. (2018). Mechanisms in Endocrinology: SGLT2 inhibitors: Clinical benefits by restoration of normal diurnal metabolism?. Eur. J. Endocrinol..

[105] Marathias K.P., Lambadiari V.A., Markakis K.P., Vlahakos V.D., Bacharaki D., Raptis A.E., Dimitriadis G.D., Vlahakos D.V. (2020). Competing Effects of Renin Angiotensin System Blockade and Sodium-Glucose Cotransporter-2 Inhibitors on Erythropoietin Secretion in Diabetes. Am. J. Nephrol..

[106] Sano M. (2018). A new class of drugs for heart failure: SGLT2 inhibitors reduce sympathetic overactivity. J. Cardiol..

[107] Palygin O., Spires D., Levchenko V., Bohovyk R., Fedoriuk M., Klemens C.A., Sykes O., Bukowy J.D., Cowley A.W., Lazar J. (2019). Progression of diabetic kidney disease in T2DN rats. Am. J. Physiol. Ren. Physiol..

[108] Jaikumkao K., Pongchaidecha A., Chatsudthipong V., Chattipakorn S.C., Chattipakorn N., Lungkaphin A. (2017). The roles of sodium-glucose cotransporter 2 inhibitors in preventing kidney injury in diabetes. Biomed. Pharmacother..

[109] Kanasaki K., Kawakita E., Koya D. (2019). Relevance of Autophagy Induction by Gastrointestinal Hormones: Focus on the Incretin-Based Drug Target and Glucagon. Front. Pharmacol..

[110] Packer M. (2020). Interplay of adenosine monophosphate-activated protein kinase/sirtuin-1 activation and sodium influx inhibition mediates the renal benefits of sodium-glucose co-transporter-2 inhibitors in type 2 diabetes: A novel conceptual framework. Diabetes Obes. Metab..

[111] Korbut A.I., Taskaeva I.S., Bgatova N.P., Muraleva N.A., Orlov N.B., Dashkin M.V., Khotskina A.S., Zavyalov E.L., Konenkov V.I., Klein T. (2020). SGLT2 Inhibitor Empagliflozin and DPP4 Inhibitor Linagliptin Reactivate Glomerular Autophagy in db/db Mice, a Model of Type 2 Diabetes. Int. J. Mol. Sci..

[112] Packer M. (2020). Autophagy-dependent and -independent modulation of oxidative and organellar stress in the diabetic heart by glucose-lowering drugs. Cardiovasc. Diabetol..

[113] Zhou H., Wang S., Zhu P., Hu S., Chen Y., Ren J. (2018). Empagliflozin rescues diabetic myocardial microvascular injury via AMPK-mediated inhibition of mitochondrial fission. Redox Biol..

[114] Greco E.V., Russo G., Giandalia A., Viazzi F., Pontremoli R., De Cosmo S. (2019). GLP-1 Receptor Agonists and Kidney Protection. Medicina.

[115] Larsen P.J., Fledelius C., Knudsen L.B., Tang-Christensen M. (2001). Systemic administration of the long-acting GLP-1 derivative NN2211 induces lasting and reversible weight loss in both normal and obese rats. Diabetes.

[116] Carraro-Lacroix L.R., Malnic G., Girardi A.C. (2009). Regulation of Na+/H+ exchanger NHE3 by glucagon-like peptide 1 receptor agonist exendin-4 in renal proximal tubule cells. Am. J. Physiol. Ren. Physiol..

[117] Yang S., Lin C., Zhuo X., Rao S., Xu W., Cheng Y., Yang L. (2020). Glucagon-like peptide-1 alleviates diabetic kidney disease through activation of autophagy by regulating AMP-activated protein kinase-mammalian target of rapamycin pathway. Am. J. Physiol. Endocrinol. Metab..

[118] Lopaschuk G.D., Verma S. (2020). Mechanisms of Cardiovascular Benefits of Sodium Glucose Co-Transporter 2 (SGLT2) Inhibitors: A State-of-the-Art Review. JACC Basic Transl. Sci..

[119] He Q., Sha S., Sun L., Zhang J., Dong M. (2016). GLP-1 analogue improves hepatic lipid accumulation by inducing autophagy via AMPK/mTOR pathway. Biochem. Biophys. Res. Commun..

[120] Sugahara M., Pak W.L.W., Tanaka T., Tang S.C.W., Nangaku M. (2021). Update on diagnosis, pathophysiology, and management of diabetic kidney disease. Nephrology.

[121] Kaushal G.P., Chandrashekar K., Juncos L.A., Shah S.V. (2020). Autophagy Function and Regulation in Kidney Disease. Biomolecules.

[122] Qi Y.Y., Zhou X.J., Cheng F.J., Hou P., Ren Y.L., Wang S.X., Zhao M.H., Yang L., Martinez J., Zhang H. (2018). Increased autophagy is cytoprotective against podocyte injury induced by antibody and interferon in lupus nephritis. Ann. Rheum. Dis..

[123] Bhayana S., Baisantry A., Kraemer T.D., Wrede C., Hegermann J., Bräsen J.H., Bockmeyer C., Ulrich Becker J., Ochs M., Gwinner W. (2017). Autophagy in kidney transplants of sirolimus treated recipients. J. Nephropathol..

